# Monensin Inhibits Triple-Negative Breast Cancer in Mice by a Na^+^-Dependent Cytotoxic Action Unrelated to Cytostatic Effects

**DOI:** 10.3390/cells14030185

**Published:** 2025-01-25

**Authors:** Simone Fiorilla, Francesco Tasso, Nausicaa Clemente, Tommaso Trisciuoglio, Renzo Boldorini, Rita Carini

**Affiliations:** Department of Health Science, Università del Piemonte Orientale, Via Solaroli, 17, 28100 Novara, Italy; simone.fiorilla@uniupo.it (S.F.); francesco.tasso@uniupo.it (F.T.); nausicaa.clemente@med.uniupo.it (N.C.); tommaso.trisciuoglio@uniupo.it (T.T.); renzo.boldorini@med.uniupo.it (R.B.)

**Keywords:** Na^+^ ionophores, cancer cell death, anticancer treatment

## Abstract

Triple-negative breast cancer (TNBC) represents the most aggressive breast cancer subtype, defined by its limited therapeutic options and poor outcomes. This study investigated the therapeutic potential of targeting Na^+^ homeostasis in TNBC cells to induce TNBC inhibition. For this purpose, BALB/c mice were inoculated with 4T1-Luc2 breast cancer cells and treated with the Na^+^ ionophore monensin (8 mg/kg) or vehicle alone. Tumor development and cellular Na^+^ content were assessed using vivo live imaging techniques, while intracellular Na^+^ variations and cytotoxicity were evaluated through live cell analysis. Monensin treatment increased Na^+^ levels in cancerous tissues and reduced TNBC mass (monensin: 0.146 ± 0.06; vehicle: 0.468 ± 0.2 cm^3^; *p* < 0.001). This treatment induced extensive necrosis in TNBC tumors while preserving the structural and functional integrity of healthy organs and maintaining the proliferative activity of both tumor and normal tissues. Monensin did not alter the expression of proliferating nuclear antigen (PCNA) in 4T1-Luc2 cells but triggered cytotoxicity preceded by intracellular Na^+^ accumulation. Na^+^-free conditions prevented both Na^+^ accumulation and 4T1-Luc2 cell death. Thus, monensin exerts its antitumor effects in TNBC through a Na^+^-dependent and tumor-specific cytotoxic mechanism, without inducing cytostatic effects on normal or transformed tissues. Collectively, these findings underscore the potential of Na^+^ ionophores as promising therapeutic agents for TNBC.

## 1. Introduction

Breast cancer is the most prevalent malignancy globally, with triple-negative breast cancer (TNBC) accounting for 15–20% of all diagnoses [1]. TNBC is characterized by the absence of estrogen and progesterone receptors, along with human epidermal growth factor receptor 2 (HER2) [2], making it unresponsive to hormone and HER2-targeted therapies that benefit other breast cancer subtypes [3]. As a result, treatment options for TNBC are largely confined to surgery and chemotherapy. Nonetheless, the high recurrence rates and limited overall survival (OS) associated with these approaches make the development of more effective therapeutic strategies a pressing priority [4,5].

This unmet clinical need is even more apparent if we consider that current advancements with poly (ADP-ribose) polymerase (PARP) inhibitors and immune checkpoint (IC) inhibitors show limited effectiveness in TNBC, beneficial only to specific patient subsets [6]. Indeed, only 15–25% of TNBC cases harbor germline BRCA1/2 (gBRCA) mutations [7], and PD-L1 protein is detected in about one-fifth of all TNBC patients [8]. Furthermore, while these therapies demonstrate efficacy in early-stage TNBC, their impact on advanced disease remains largely insufficient [6].

A hallmark of cancer metabolism is the shift from mitochondrial to aerobic glycolysis, a phenomenon known as the Warburg effect [9]. This metabolic adaptation leads to the accumulation of acidic glycolytic byproducts, yet cancer cells maintain an alkaline intracellular pH [10]. This apparent contradiction is explained by the increased activity or expression of pH-regulatory proteins, many of which are Na^+^ transporters that induce a net sodium entry into the cell [10]. Early studies from our group showed that mitochondrial dysfunction in primary, non-transformed rodent hepatocytes, triggers an irreversible rise in intracellular Na^+^ levels which, altering the mechanisms of cell volume maintenance, causes hepatocyte death. Congruently, preventing this Na^+^ accumulation conferred enhanced resistance to cellular damage [11,12,13].

Based on these observations, we hypothesize that TNBC cancer cells, by inherently containing elevated intracellular Na^+^ levels compared with normal cells, would be energetically unable to compensate for further increases in Na^+^ concentration induced by pharmacological treatment with sodium ionophore compounds.

Consistently, our recent research on a mouse model of HCC [14] demonstrated that liver cancer tissues exhibited significantly elevated levels of intracellular Na⁺ compared with normal tissues, a disparity that was further exacerbated by the treatment with the antibiotic cation ionophore monensin [14]. Monensin selectively killed HCC cells and not healthy hepatocytes [14] by inducing sodium-dependent mitochondrial alterations, ATP loss, and energy-dependent inhibition of the Na^+^/K^+^ pump with a consequent irreversible increase in intracellular sodium. This condition of permanent energetic stress made HCC cells unable to keep, unlike healthy hepatocytes, life-compatible levels of Na^+^ with a consequent increase in the cellular osmotic pressure and death of the cancer cells [14].

This research was the first to demonstrate that cancer cells are acutely sensitive to a pharmacologically induced Na^+^ overload via monensin.

The finding that the antibiotic monensin possessed a potent anticancer activity had been, however, previously established by several other studies [15,16,17,18,19]. Monensin has been, in fact, shown to inhibit cell proliferation and induce apoptosis across different cancer cell types, including those exhibiting multidrug resistance [15] and breast cancer cells [19]. Such effects were found to be associated with the inhibition of signal mediators involved in the regulation of cell division and apoptosis, such those of the Wnt/β-Catenin, EGF receptor, or SUMO signal pathway [15,16,17,18,19]. In the specific case of the breast cancer cells, monensin was reported to induce cytotoxicity and repress colony cell formation by inhibiting a component of the SUMO pathway, UBA2 [19].

None of these previous studies, however, linked the anticancer activity of monensin to its primary chemical property as a cation ionophore [20]. Monensin is, in fact, a polyether cation ionophore able to reversibly tie and transport Na^+^ from the extracellular liquid ([Na^+^]e ≃ 145 mM) to the cytoplasm ([Na^+^]i ≃ 15 mM) along its concentration slope [20].

Aerobic glycolytic metabolism and alkaline intracellular pH are nearly ubiquitous features of cancer tissues, with increased intracellular Na^+^ levels likely arising as a consequence of these metabolic conditions [9,10]. Significantly, this relationship has been substantiated by recent 23Na-magnetic resonance imaging analyses of various human cancers [21,22,23,24,25,26], including breast cancer [24,25], that consistently identified aberrantly high intracellular Na^+^ levels in cancerous tissues relative to adjacent normal tissues. These findings not only reveal Na^+^ concentration as a novel diagnostic biomarker for human tumors [26] but also provide a compelling scientific base for the hypothesis that triple-negative breast cancer (TNBC) could be specifically sensitive to a further sodium accumulation and that TNBC cells would be specifically killed upon exposure to the sodium ionophore monensin, thus inducing TNBC inhibition by a mechanism unrelated to cytostatic effects.

The present study investigated these issues, exploring, for the first time in a mouse in vivo model, the anticancer potential of monensin against TNBC by focusing on its ability to influence Na^+^ dynamics and cell viability and analyzing the possible conditions responsible for the inconsistencies of the results regarding the cytostatic or cytotoxic effects of monensin.

To this purpose, using both in vitro and in vivo imaging approaches, we measured Na^+^ accumulation and cancer cell death in murine TNBC cells (4T1-Luc2), alongside tumor mass reduction in mice injected with 4T1-Luc2 cells and intraperitoneally (i.p.) with monensin. We developed a novel methodology able, unlike the available techniques, to estimate in vivo the intracellular variations of the sodium level and explored the effects of monensin on cancer and healthy tissues, focusing on markers of proliferation.

Our findings confirmed that monensin selectively induced massive necrosis in TNBC tumors through a sodium-dependent mechanism without affecting the proliferative activity of transformed and normal tissues, thus distinguishing its cytotoxic from cytostatic effects as also enlightened by the role of differential in vitro settings in producing the discrepancies regarding its mechanism of action.

Collectively, these results underscore the potential of targeting Na⁺ homeostasis with sodium ionophores like monensin as innovative therapeutic agents for TNBC able to specifically kill TNBC cells without altering healthy tissues.

## 2. Materials and Methods

### 2.1. Chemicals and Reagents

Fetal bovine serum (FBS), penicillin (P), streptomycin (S), monensin, trypan blue, 2-hydroxypropyl-β-cyclodextrin (HBCD), acrylamide/bis-acrylamide (30% solution), Amersham Hybond PVDF Blotting Membrane, anti-PCNA (Ab-1) mouse mAb (PC10) 1:100, monoclonal anti-β-actin mouse antibody 1:1000, and all chemicals for buffer and reagent preparations were obtained from Sigma-Aldrich (MO, USA). Pierce^TM^ RIPA buffer, Novex^TM^ tris-glycine transfer buffer, Pierce^TM^ BCA protein assay, Pierce^TM^ ECL Western blotting substrate, and all chemicals for buffer and reagent preparations for Western blot analysis were purchased from Thermo Fisher Scientific (MA, USA). Roswell Park Memorial Institute medium (RPMI), Dulbecco’s modified eagle medium (DMEM), and custom-made Na^+^-free DMEM (DMEM-Na^+^), with no Na^+^ constituents, were obtained from GIBCO (S.I.A.L. group, Rome, Italy). ION NaTRIUM Green-AM was obtained from Abcam (Cambridge, UK), and CoroNa™ Green, AM, was from Thermo Fisher Scientific (MA, USA). Ultra Rediject salt D-luciferin was obtained from Perkin Elmer (MA, USA).

### 2.2. Cell Culture

Murine 4T1-Luc2-Luc2 (4T1-Luc2) mammary tumor cells, murine B16-F10-Luc (B16) melanoma tumor cells, human SK-OV-3 (SKOV3) ovarian tumor cells, and murine Hepa-1Hepa-C1C7 (HEPA-C1C7) were supplied by LGC Ltd. (Middlesex, UK). Human MCF7 mammary tumor cells were obtained from American Type Culture Collection (ATCC; Manassas, VA, USA).

The 4T1-Luc2, B16, and SKOV3 cells were routinely cultured in RPMI supplemented with 10% FBS, 100 U/mL penicillin, 100 μg/mL streptomycin. HEPA-C1C7 and MCF7 cells were cultured in DMEM without glutamine, added with 10% FBS, 100 U/mL penicillin, 6 mM glutamine, and 100 μg/mL streptomycin (all materials were from Lonza Bioscience, Basel, Switzerland). Cells were kept under 5% CO_2_/95% air atmosphere at 37 °C as previously described [14]. All cell lines were tested for mycoplasma contamination using the MycoAlert™ mycoplasma detection kit.

### 2.3. Cell Treatments

For in vitro cell imaging analysis or for the measurement of cell viability, ATP, and Na^+^ content, 4T1-Luc2 or MCF7 cells were seeded (1.5 × 10^5^/mL cell density) in RPMI and treated with 1 μM monensin (or vehicle) after 24 h in DMEM with normal (145 mM) sodium concentration (+Na^+^) or without any sodium component (−Na^+^) (GIBCO, S.I.A.L, Rome, Italy) in the absence of serum.

For WB analysis, 4T1-Luc2-Luc2 (4T1-Luc2), B16, SKOV3, and HEPA-C1C7 cells were seeded (1.25 × 10^5^/mL cell density) in RPMI or DMEM (HEPA-C1C7) and treated with 5 μM monensin (or vehicle) after 16–24 h in DMEM/RPMI supplemented or not with fetal bovine serum (FBS, 10%).

### 2.4. Cell Viability, ATP, and Na^+^ Content

Cell viability and intracellular ATP were determined by using, respectively, the CellTox and Cell-Titer protocols (Promega; Madison, WI, USA) as previously reported [14]. The ATP values were corrected excluding ATP released by dead cells, as determined by simultaneous CellTox and Cell-Titer analyses performed for each sample. Viability and ATP values were expressed as a % of corresponding controls.

The 4T1-Luc2-Luc 2 and MCF7 cells were loaded with the intracellular Na^+^-fluorescent dye ION NaTRIUM Green-AM (5 μM) in Hank’s Balanced Salt Solution (HBSS) with BSA (2%), pluronic acid (10 μM), and glucose (10 mM) for 1 h at RT. Intracellular Na^+^ variations upon monensin treatment were monitored using a Kontron SFM25 spectrofluorometer set at 525 nm excitation and at 545 nm emission wavelengths and expressed as a % of control values.

### 2.5. In Vitro Cell Imaging Analysis of CoroNa Green, AM/Cytotox NIR Dye Fluorescence

The 4T1-Luc2 cells were seeded in a 96-well plate for 24 h in DMEM supplemented with 10% FBS and subsequently loaded with the fluorescent dye CoroNa Green-AM. This dye acts as a sodium ion indicator requiring intracellular de-esterification to bind Na^+^, with Na^+^ binding leading to an increase in green fluorescence emission intensity with minimal wavelength shift. The 4T1-Luc2 cells were incubated with the fluorescent probe (5 μM) in HBSS containing BSA (4%) and pluronic acid (0.02%) at 37 °C for 1 h and then washed twice with HBSS. Cells were then exposed to monensin (1 μM) and the cell-impermeant cytotox NIR dye (0.6 μM) to visualize the nuclei of dead cells and maintained for 20 h in DMEM in the presence or absence of Na^+^. Intracellular Na^+^ variations were recorded every 3 min, while cytotoxicity was assessed hourly using a semi-automated analysis protocol of the IncuCyte SX5 Live^®^ Cell Analysis System. The Sartorius^®^ probe in the same imaging system facilitated pre-set parameters for optimal fluorescent outputs.

The in vitro (Incucyte) fluorescence intensity data were collected from the Sartorius Incucyte 2022B Rev2 software and analyzed through the GraphPad Prism 8 software. Raw fluorescence intensity data, measured by the instrument as total green object integrated intensity (GCU × um^2^/Image) or as total NIR object integrated intensity (NIRCU × um^2^/Image), were used for the graphs. The intracellular sodium probe (CoroNa Green-AM; max Ex.: 492 nm, max Em.: 516 nm) was captured by using the default green channel (Ex.: 453–485 nm, Em.: 494–533 nm), while the cytotoxicity probe (IncuCyte^®^ Cytotox NIR Dye; max Ex.: 665 nm, max Em.: 695 nm) was captured by using the default near-infrared channel (Ex.: 648–674 nm, Em.: 685–756 nm). All the images were acquired through the included 20× (0.62 um/px) objective. The in vivo (IVIS Spectrum) fluorescence intensity data were collected from and analyzed through the Living Image ® 4.7.4 software (Revvity). Raw fluorescence intensity data, measured by the instrument as p/sec/cm^2^/sr and analyzed through the Living Image™ software, were used for the graphs. The luciferin images were captured using the protocol provided by the producer (Bioluminescence, open filter, automatic exposure), while the intracellular sodium probe was captured by using a 500/40 fluorescence filter and automatic exposure.

### 2.6. Quantitative Expression of Proliferating Cell Nuclear Antigen (PCNA)

Protein extracts from 4T1-Luc2, HEPA-C1C7, B16, and SKOV3 cells were electrophoresed by SDS/PAGE (10% gel), and after blotting them onto the PVDF membranes, the membranes were probed with antibodies against PCNA ( Sigma-Aldrich (MO, USA)). The β-actin monoclonal antibody (Sigma-Aldrich (MO, USA ) was used to assess equal protein loading. The antigens were detected by ECL Western Blotting Substrate (Thermo Fisher Scientific, MA, USA) and a ChemiDoc MP quantitative imaging system (BioRad Laboratories, Milan, Italy). The results were expressed as ratios.

### 2.7. In Vivo Experiments

The 4T1-Luc2-Luc2 tumor cells (100,000 cells in 100 μL of 0.9% NaCl per mouse) were orthotopically injected into the fourth mammary fat pad of female BALB/c mice aged six to eight weeks (Envigo, Inc., IN, USA). Tumor growth was monitored daily, and when the tumor size reached approximately 80 mm^3^, the mice were treated daily with intraperitoneal (i.p.) injections of 4, 8, or 12 mg/kg monensin, dissolved in DMSO/HBCD 10% in 0.9% NaCl (1:9, *v/v*) or with vehicle alone (DMSO/HBCD 10% in 0.9% NaCl–1:9, *v/v*). Groups of five (treatment with 4 or 12 mg/kg monensin) or fifteen animals (treatment with 8 mg/kg Momensin or vehicle) were employed. Tumors were measured in two dimensions using an analog caliper to estimate the tumor volume using the formula V = (L × W × W)/2.

Treatments were administered for 10 days, and mice were sacrificed 2 h after the last administration or upon signs of distress.

### 2.8. In Vivo Imaging Analysis of Tumor Development and Intratumoral Na^+^ Content

Bioluminescence imaging using the IVIS^®^ Spectrum was employed to monitor the distribution of 4T1-Luc2-Luc2 tumor cells and the development of the tumor. The IVISbrite™ D-luciferin ultra bioluminescent substrate in RediJect™ solution (RediJect D-luciferin ultra K^+^ salt) was injected i.p. following the manufacturer’s guidelines (150 mg/kg). The bioluminescence signal, derived from living luciferase-expressing cells, directly represented the tumor mass, whereas a decrease in bioluminescence signals corresponded to necrotic areas. The fluorescent cytosolic Na^+^ indicator CoroNa™ Green-AM was used to assess the Na^+^ concentrations in tumor and healthy tissues. Just prior to imaging acquisition, mice were injected with a solution (100 μL) containing the intracellular Na^+^-fluorescent dye (67 μM) in 0.9% NaCl with BSA (4%) and pluronic acid (0.02%). Intracellular Na^+^ variations following monensin treatment were monitored using the IVIS^®^ Spectrum in vivo imaging system set to excitation at 500 nm and emission at 540 nm.

### 2.9. Histology and Immunohistochemistry

Mouse tissues were routinely embedded in paraffin and fixed in 10% neutral buffered formalin. Bone marrow samples were decalcified for 8 h using an EDTA-based decalcifying solution, washed in running water for 1 h, and subsequently embedded in paraffin. Four-micron-thick sections were deparaffinized, rehydrated, and stained with hematoxylin and eosin for histopathologic analyses or with anti-Ki-67 (1:250, Ventana^®^ Medical Systems, Roche, Monza, Italy) to evaluate cell proliferation by using an automated immunostainer (Ventana, Roche, Monza, Italy). Unstained or corresponding hematoxylin and eosin-stained sections were used as controls of anti-Ki-67-stained samples. Slide images were captured using the ZEISS Axioscan 7 slide scanner (objective: Plan-Apochromat 40x/0.95 corr M27; camera: ZEISS Axiocam 705 color). Ki-67-positive cells, cell density (cells/mm^2^), and cell size (EQPC: equivalent diameter of a circle with the same projection area as the cell) were quantified using QuPath’s built-in “Positive cell detection” and “Cell detection” tools (software version QuPath-0.5.1).

### 2.10. Statistics and Reproducibility

All experiments were independently repeated at least three times. The results were presented as the mean of three to eight independent experiments ± standard deviation. Statistical significance between two groups was determined by ordinary two-way ANOVA with Sidak’s multiple comparisons test. Data analysis was performed using GraphPad Prism 8.4.3 (GraphPad Software, Inc., La Jolla, CA, USA).

## 3. Results

### 3.1. Monensin Exerts Cytotoxic and Na^+^-Dependent Effects on TNBC Cells Without Affecting PCNA Expression

The anticancer activity of monensin was evaluated in TNBC cells maintained in oxygenated conditions in normal DMEM medium or custom-modified Na^+^-free DMEM medium in the absence of FBS by Cell Tox analysis. As already reported [14], treatments were performed in the absence of FBS to better simulate the in vivo physiological environment where cells of solid tumors are minimally exposed to serum proteins, except theoretically or in trace amounts [27]. 

**Figure 1:**
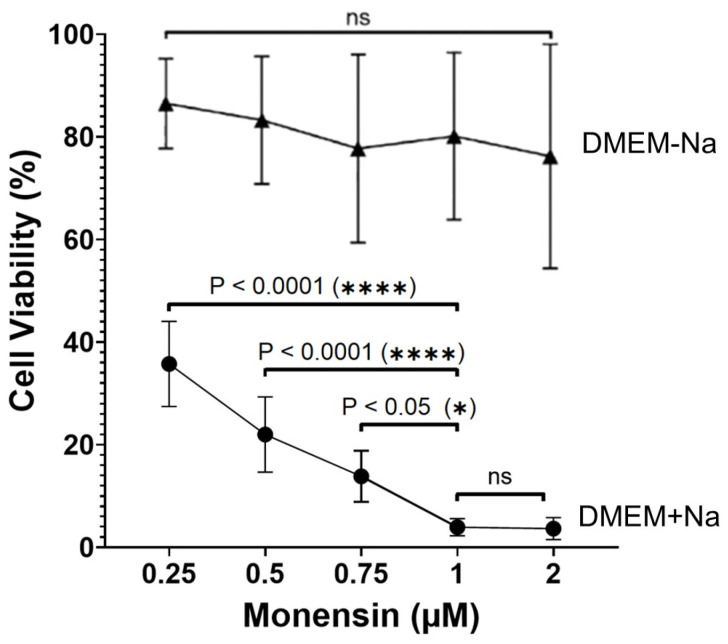
Dose-dependent analysis of the cytotoxic effects of monensin on 4T1-Luc2-Luc2 cells in DMEM ± Na^+^. Effects of 24 h treatment with 0.25, 0.5, 0.75, 1, and 2 µM monensin on 4T1-Luc2-Luc2 cells viability. Results are expressed as % controls (n = 3 independent experiments). Round symbols, monensin DMEM + Na^+^; triangular symbols, monensin DMEM−Na^+^. Significance was evaluated with O-way analysis of variance (ANOVA) Bonferroni multiple comparisons test. ns = not significant.

We examined the effects of monensin on the cell viability of the murine TNBC 4T1-Luc2 cells by Cell Tox analysis. As shown in Figure 1, monensin killed, in a dose-dependent manner, 4T1-Luc2 cells maintained in DMEM medium for 24 h with a maximum effect observed at 1 μM concentration, and the incubation in the Na^+^-free DMEM medium prevented the appearance of cell damage.

**Figure 2:**
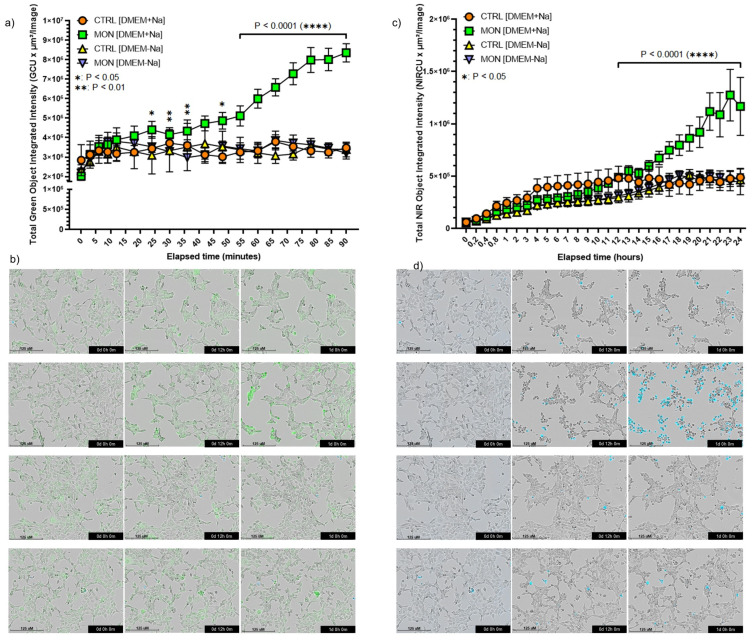
Monensin increases sodium content and induces a sodium-dependent cytotoxic effect in 4T1-Luc2-Luc2 cancer cells. (**a**) Effects of a 90 min treatment with 1 µM monensin on the intracellular Na^+^ content of 4T1-Luc2-Luc2 cells. The grouped superimposed plot displays the integrated intensity of the sodium probe in DMEM with or without Na^+^. (**b**) Representative in vivo cell images showing intracellular Na^+^ increase in 4T1-Luc2 cells exposed to monensin in DMEM with or without Na^+^. (**c**) Cytotoxic effects of a 24 h treatment with 1 µM monensin on 4T1-Luc2-Luc2 cells. The grouped superimposed plot shows the positivity to the cytotoxicity probe in DMEM with and without Na^+^. (**d**) Representative in vivo cell images depicting cell death in 4T1-Luc2 cells exposed to monensin in DMEM with or without Na^+^. Data are presented as mean ± SD (n = 6) and statistically analyzed using a two-way ANOVA and were considered statistically significant when * *p* < 0.05.

We next employed the Incucyte SX5 live-cell analysis system to determine by cell in vivo imaging the effects of monensin 1 μM on intracellular sodium content and cell damage of 4T1-Luc2_luc2 cells loaded with the fluorescent sodium dye CoroNa Green-AM and co-stained with Cytotox NIR. As shown in Figure 2a, monensin treatment led to a sustained and significant increase in intracellular Na^+^ levels observable as early as after 30 min of exposure (Figure 2a) and also evident after 12 and 24 h of exposure to the ionophore (Figure 2b). Cell damage became instead evident starting from 12 h of incubation with a maximum effect at 24 h (Figure 2c,d).

These effects were entirely abrogated in cells maintained in Na^+^-free DMEM medium, confirming the results obtained by Cell Tox analysis (Figure 1). These data also showed that the increase in sodium intracellular content preceded the appearance of cell death and indicated that the cytotoxic action of monensin depended on an influx of sodium from the extracellular medium.

CellTox and cell-titer analyses of ATP and the viability of murine 4T1-Luc2 and human MCF7 TNBC cells and the evaluation of intracellular Na^+^ content of the same cells loaded with ION NaTRIUM Green-AM provided evidence that the absence of sodium in the incubation medium prevented the increase in intracellular Na^+^ and the decrease in ATP content induced by 8 h treatment of cells with monensin (1 µM), as well as the loss of viability induced by 24 h treatment (Table 1). This showed that, in agreement with what was previously observed with HCC cells [14], monensin exerted in both human and mice TNBC cells a Na^+^-dependent cytotoxic action that was preceded by the increase in cellular sodium content and by a sodium-dependent depletion of energy disposal.

**Figure 3:**
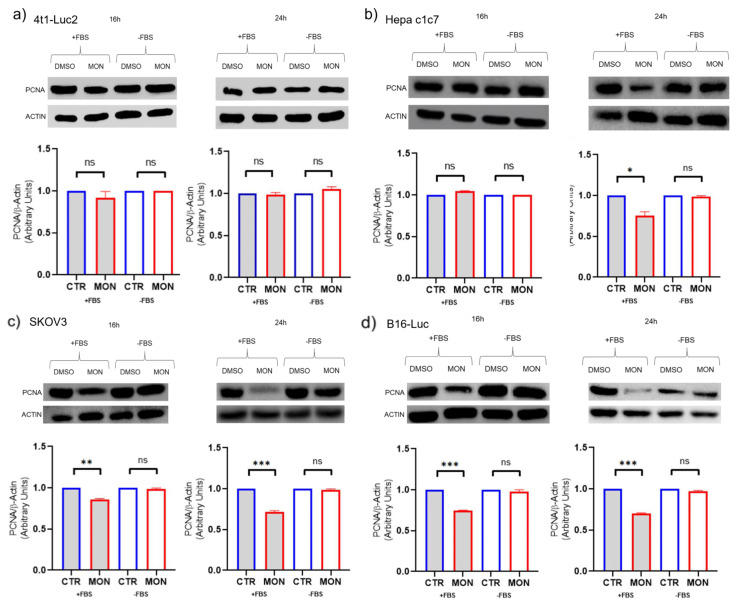
PCNA protein expression in monensin-treated cells. Western blot analysis and densitometric quantifications showing actin-normalized PCNA protein levels of 4T1-Luc2-Luc (4T1-Luc2) (**a**), Hepa HEPA-C1C7 (HEPA-C1C7) (**b**), SKOV3 (**c**), and B16-Luc (B16) (**d**) cells treated with 5 µM monensin (MON) or vehicle (CTR) in the presence or absence of fetal bovine serum (FBS) for 16 and 24 h. Data are presented as mean ± SD (n = 3) and statistically analyzed. * *p* < 0.05, ** *p* < 0.01, *** *p* < 0.001, ns = not significant, with unpaired *t*-test and Welch’s correction.

The effect of monensin on the protein expression levels of the proliferative marker PCNA of 4T1-Luc2-Luc2 cells was evaluated in comparison with that exerted on murine hepatocarcinoma Hepa-C1C7, murine melanoma B16, and human ovarian cancer SKOV-3 cells. PCNA expression was assessed following 16 or 24 h of monensin exposure, with or without 10% FBS in the medium. The treatment condition including FBS was applied to replicate experimental settings from previous studies demonstrating the cytostatic activity of monensin [19,20,21,22,23,24,25,26]. As shown in Figure 3a, no significant differences in PCNA expression were observed in 4T1-Luc2-Luc2 cells treated with monensin or vehicle, irrespective of serum presence. On the other hand, monensin treatment reduced PCNA expression of Hepa-C1C7 cells at 24 h (Figure 3b) and in SKOV-3 and B16 cells at both 16 and 24 h when maintained in the presence of FBS (Figure 3c and 3d, respectively). In contrast, under serum-free conditions, monensin had no impact on PCNA expressions in any of the cell lines tested. This indicated that the effect of monensin on PCNA expression was both cell-type specific and dependent on the presence of serum. This showed that certain cell types could present no (4T1-Luc2-Luc2 cells) or delayed (Hepa-C1C7 cells) sensitivity to the serum-dependent pro-proliferative signals affected by monensin and also provided evidence that in the absence of such exogenous conditions (exposure to FBS), monensin was unable to modify the endogenous capacity to express the proliferation marker in all the cell types.

### 3.2. Monensin Inhibits TNBC Growth and Increases Intracellular Na^+^ in Tumor Cells

**Figure 4:**
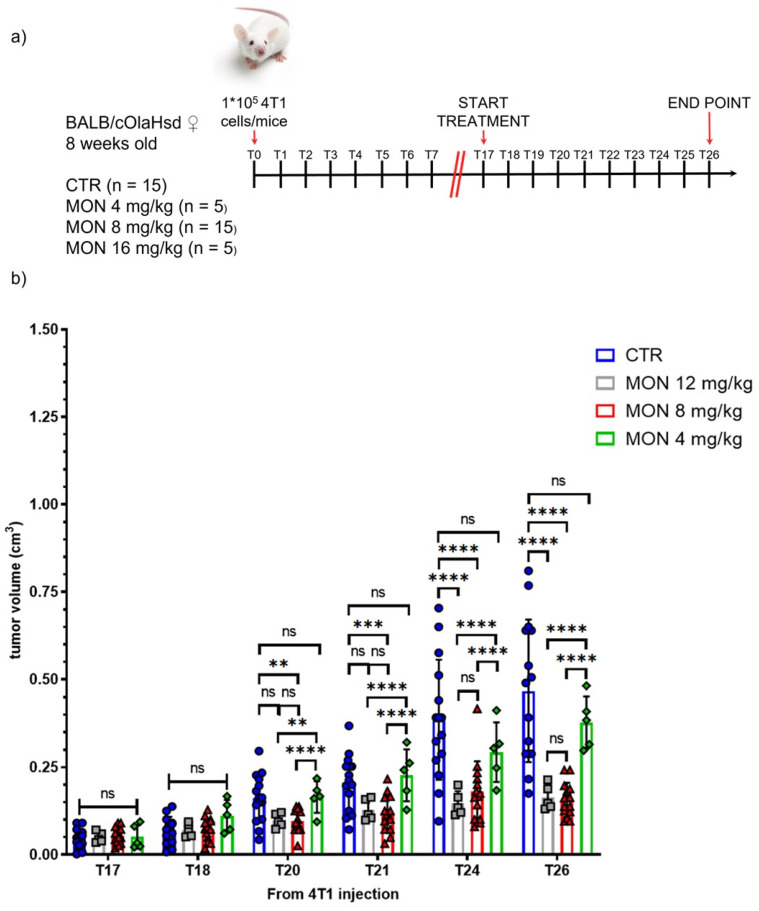
Systemic administration of monensin (MON) inhibits the development of triple-negative breast cancer (TNBC). (**a**) Experimental timeline of allograft tumor transplantation and treatment. (**b**) Growth of allograft tumors of 4T1-Luc2 cells in BALB/c mice treated i.p. with monensin (MON) or vehicle (CTR). Data are presented as mean ± SD and statistically analyzed using a two-way ANOVA. ** *p* < 0.01, *** *p* < 0.005, **** *p* < 0.001, ns = not significant.

TNBC tumor allograft was obtained by orthotopic injection of 4T1-Luc2-Luc2 tumor cells into the fourth mammary fat pad of female BALB/c mice. The anticancer activity of monensin was evaluated through daily i.p. administration of this ionophore at the dosages of 4, 8, or 12 mg/kg/day starting on day 17 (Figure 4a) post-implantation of the TNBC cells, and tumor growth was monitored in comparison with those of mice receiving vehicle alone.

As shown in Figure 4b, monensin treatment resulted in a marked reduction in TNBC tumor size with significant effects at the doses of 8 and 16 mg/kg/day. The dosage of 8 mg/kg/day was thus selected for the subsequent analysis.

**Figure 5:**
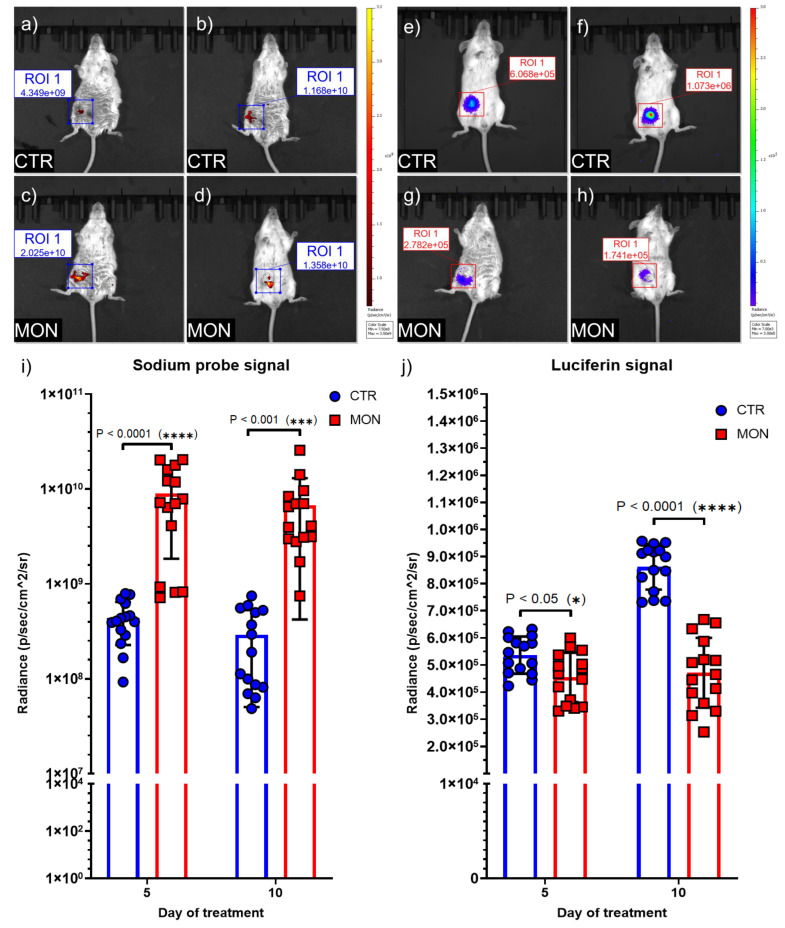
Monensin reduces TNBC growth and elevates intracellular sodium levels in tumor cells. (**a**–**h**) TNBC IVIS^®^ Spectrum imaging was used to monitor tumor growth and tissue Na^+^ in BALB/c mice bearing 4T1-Luc2-Luc2 breast tumors treated with monensin (8 mg/kg) (MON) or vehicle (CTR). Images were captured following luciferin injection with auto-exposure length and an open filter and after intraperitoneal injection of CoroNa™ Green-AM solution with auto-exposure length and 500/40 fluorescence filters. The images in panels (**a**,**c**,**e**,**g**) were taken on the 5th day of treatment, while the images in panels (**b**,**d**,**f**,**h**) were taken on the 10th (final) day of treatment. Radiance values are expressed in photons per second per square centimeter per steradian (p/s/cm²/sr). (**i**,**j**) The average signals from the sodium probe (**i**) and luciferin (**j**) for each group (n = 15 control, n = 15 MON-treated mice) at different time points were calculated using the IVIS^®^ Spectrum Living Image analysis software. Data are presented as mean ± SD and were statistically analyzed using a two-way ANOVA. * *p* < 0.05, *** *p* < 0.001, **** *p* < 0.0001.

In vivo imaging using the IVIS Spectrum was performed after 5 and 10 days of monensin treatment to evaluate intracellular Na^+^ levels (Figure 5a–d), as well as tumor extension (Figure 5e–h). A reduction in luciferin signal intensity in monensin-treated mice confirmed tumor mass shrinkage (Figure 5g–j), consistent with caliper measurements. Furthermore, the CoroNa™ Green-AM fluorescence indicated elevated Na^+^ concentration in TNBC tumors in monensin-treated mice (Figure 5c,d,i), with no detectable signal in the surrounding healthy tissues. For these experiments, we developed the conditions (see Material and Methods) needed to employ for in vivo imaging analysis the sodium probe CoroNa™ Green-AM, also used in the cell in vivo imaging analysis (Figure 3a,b). This fluorescent probe is cell-permeant, and once inside the cell, intracellular esterases cleave off the acetate moieties to convert it into the sodium-responsive form. Consequently, its employment in vivo allows visualization of sodium content of only living cells, making highly unlikely the possibility of false positives and non-specific results.

### 3.3. Monensin Promotes TNBC Necrosis Without Affecting Vital Organs or Inhibiting Proliferation in Tumor, Intestine, and Bone Marrow

**Figure 6:**
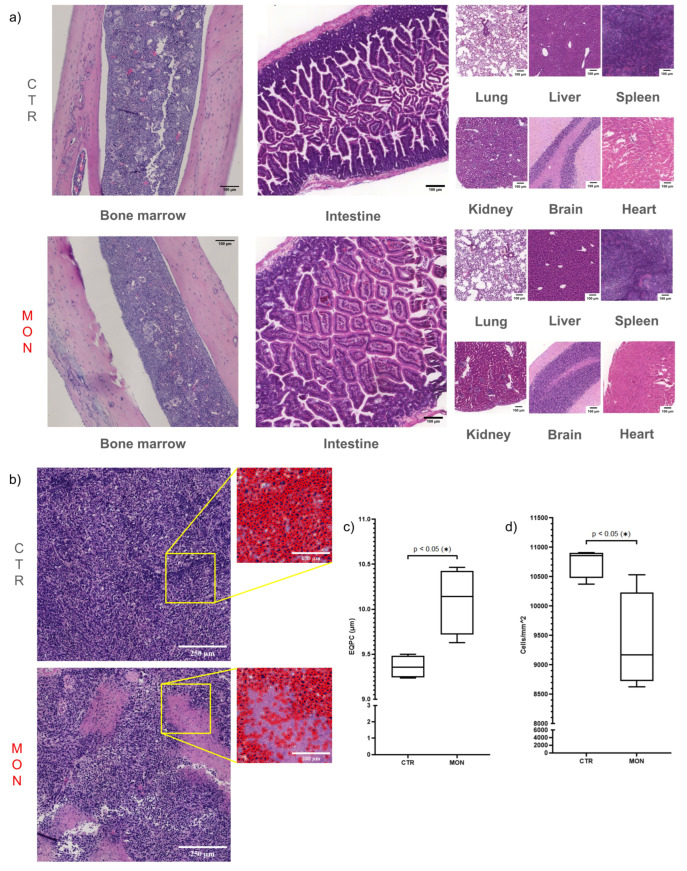
Monensin promotes TNBC necrosis without affecting vital organs. (**a**) H&E images of lung, liver, spleen, kidney, and brain of mice treated i.p. with monensin (MON) or vehicle (CTR). (**b**) H&E images of TNBC allograft of mice exposed or not to monensin. The insets show cell profiles used to evaluate cellular density and size. (**c**) Quantification of cell size as EQPC (diameter of a circle of equal projection area of the cell) in TNBC from mice treated or not with monensin. (**d**) Quantification of tumor cellular density evaluated as cells/mm^2^ in TNBC from mice treated or not with monensin. Magnification: scale bar = 250 or 100 μm * *p*  <  0.05 by unpaired *t*-test.

**Figure 7:**
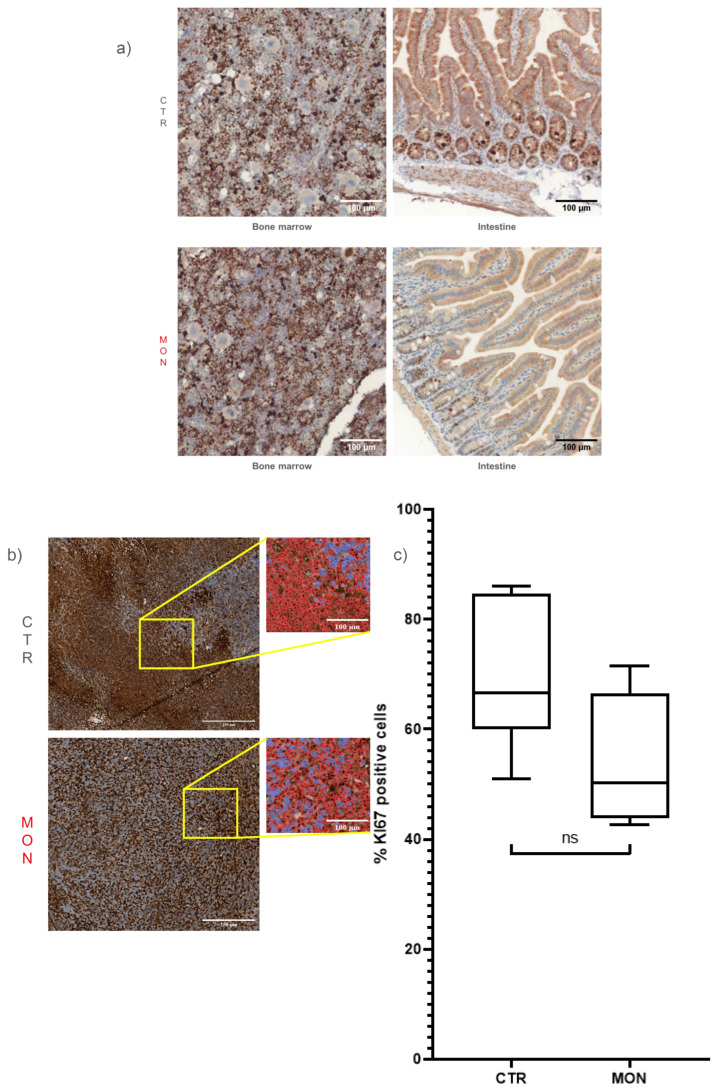
Monensin does not affect cell proliferation in tumor, intestine, and bone marrow (BM). (**a**) Representative immunohistochemical staining for Ki-67 in the intestine and bone marrow of mice treated (MON) or not (CTR) with monensin (8 mg/kg). (**b**) Representative immunohistochemical staining for Ki-67 in tumors from mice treated or not with monensin. High-resolution images show profiles of single cells outlined in red for the quantification of Ki-67-positive cells. (**c**) Plot showing the percentage of Ki-67-positive cells in the tumors of BALB/c mice treated with monensin or vehicle. Magnification: scale bar = 250 or 100 μm. Statistical test: not significant (ns) with unpaired *t*-test.

Daily administration of monensin at 8 mg/kg/day had no effect on survival, general wellness, or body weight in BAL-c mice as previously reported in NSG mice [14]. At the study endpoint, histological analyses were conducted on tumor and healthy tissues collected from sacrificed mice. Monensin treatment did not cause morphological damage in vital organs, including the lung, liver, spleen, kidney, brain, and heart, nor did it alter the structure or cellularity of the intestine and bone marrow (Figure 6a). In contrast, monensin-induced TNBC shrinkage (Figure 4b) was associated with extensive tumor necrosis, as evidenced morphologically by the presence of large pink and amorphous areas in the H&E-stained section (Figure 6b) and quantified, as previously reported [14], as a significant reduction in cellular density (Figure 6d). Consistent with previous findings in a mouse HCC model [14], monensin treatment also significantly increased the size of TNBC cells (Figure 6c). Last, monensin treatment did not affect the expression of the proliferative marker Ki-67 in either healthy proliferative tissue (i.e., intestine and BM) (Figure 7a) or TNBC specimens (Figure 7b). These findings demonstrate in vivo the lack of a cytostatic action of monensin and indicate that despite an intact proliferating activity of the TNBC cells surviving the toxic action of monensin, the massive killing and consequent loss of the TNBC cells already produced by the ionophore accounts for the shrinkage of the tumor mass detected in monensin-treated mice (Figure 4b).

Altogether, these findings provide evidence that monensin represents a selective anticancer agent that targets TNBC cells without harming vital organs or normal tissue proliferation.

## 4. Discussion

The present study investigated the anticancer effects of monensin treatment in cellular and mouse TNBC models, focusing on the ability of this ionophore to induce a specific and cytotoxic increase in intracellular Na^+^ within cancerous cells. Using live cell imaging analysis, we showed that monensin induced an early increase in intracellular Na^+^, which preceded TNBC cell death, and that maintaining cells in Na^+^-free medium effectively blocked Na^+^ upregulation, hindering monensin-induced cytotoxicity. Employing both human and mice TNBC cell types, we evidenced that the cytotoxic action of monensin was preceded by sodium-dependent energy depletion. We finally found that systemic treatment with monensin in BALB/c mice bearing orthotopic TNBC allografts led to significant TNBC tumor shrinkage and induced a further and selective increase in intracellular Na^+^ content within cancer tissues, without affecting the integrity and sodium content of healthy organs.

In this research we employed vivo imaging methodologies [28] to simultaneously and non-invasively monitor in mice TNBC development and cellular sodium variations of transformed and healthy cells, using luciferase-targeted TNBC cells along with bioluminescent and fluorescent dyes that needed intracellular processing for visualization. Using this approach, we addressed the limitations of available techniques [21,22,23,24,25], which cannot discriminate between intra- and extra-cellular Na^+^ that passively enters necrotic cells and demonstrated that the Na^+^ increase precedes cancer cell killing, excluding epiphenomena due to cancer tissue necrosis.

The results obtained were consistent with our in vitro observations in TNBC and HCC cells [14], marking the first in vivo demonstration that monensin increased the Na^+^ content of TNBC cells and that such upregulation was associated with a reduction in TNBC expansion. Our in vivo analysis was also in good agreement with findings from 23Na MRI studies on human breast cancer [20], confirming that TNBC cells displayed higher basal intracellular Na^+^ concentration ([Na^+^]i) compared with untransformed cells and indicating [Na^+^]i as a distinct and actionable target characteristic of cancer cells. Notably, we showed that monensin treatment selectively enhanced Na^+^ levels in TNBC cells without affecting healthy tissues, suggesting that unlike healthy cells, cancerous TNBC cells were unable to compensate for the monensin-driven Na^+^ load, rendering them selectively vulnerable to its cytotoxic effects.

Consistent with prior studies involving a different strain of mice (i.e., NOD.Cg-PrkdcscidIl2rgtm1Wjl/SzJ mice) and tumor type (HCC) [14], our histological analysis revealed that monensin treatment in Balb-c TNBC mice did not induce morphological damage in vital organs, such as the liver, lung, spleen, kidney, heart, and brain. By contrast, monensin-treated TNBC tumors displayed extensive areas of necrosis and pronounced swelling of tumor cells. This observation aligned with our previous findings on primary hepatocyte death showing that mitochondrial dysfunction led to an irreversible increase in intracellular Na^+^ that resulted in an abrupt increase in cellular volume with lysis and death of the cells [11,13]. Similarly, in HCC cells [14], we demonstrated that monensin triggered a Na^+^-dependent increase in intracellular water retention (k_io_) and cell enlargement within the tumors [14]. Supporting these observations, MRI and NMR relaxometry studies on human tumors [29,30,31] have also reported significantly enhanced k_io_ in cancer cells, indicative of a heightened intracellular osmotic pressure during neoplastic development.

Our findings in HCC and TNBC models, along with the evidence of elevated Na^+^ content [21,22,23,24,25] and increased intracellular water retention in human cancers [29,30,31], support the hypothesis that treatment with sodium ionophores may exacerbate these conditions, thereby inducing selective TNBC cell killing. This assumption indicated that the anti-tumor effect of monensin was primarily due to a specific cytotoxic action on tumor tissues rather than a cytostatic effect on rapidly proliferating tissues. Indeed, our previous report using an HCC allograft model provided evidence that monensin did not affect proliferative activity in either transformed or healthy tissues [14]. Consistently, the present study further confirmed in the TNBC mice model the lack of cytostatic action of monensin in both tumor and normal proliferating tissues, such as BM and intestinal mucosa. These observations are particularly significant as they support the hypothesis that monensin exerts its anti-tumor effect in TNBC without relying on a cytostatic mechanism. They are, however, in contrast with the several in vitro studies that, employing different human or murine cancer cell types, had previously linked the anti-cancer activity of monensin to its inhibitory effects on pro-proliferative pathways [15,16,17,18,19].

To investigate this question, we examined the effects of monensin on the expression of the proliferative marker PCNA in 4T1-Luc2 cells, comparing them with those observed in murine HCC cells (Hepa-C1C7), human ovarian carcinoma cells (SKOV-3), and murine melanoma cells (B16), with or without 10% FBS during incubation. The absence of serum, as used in our previous study on the cytotoxic and sodium-dependent activity of monensin in Hepa-C1C7 cells [14], was chosen to better reflect in vivo conditions where cells of solid tumors, such as breast cancer, HCC, ovarian carcinoma, and melanoma were not directly in contact with serum and, more specifically, were not exposed to the components of fetal bovine serum (FBS) [27]. Conversely, monensin treatment in the presence of FBS replicated the experimental conditions described in the articles reporting the cytostatic action of monensin [15,16,17,18,19].

Our results revealed significant differences depending on the conditions applied. Treatment with monensin in the absence of FBS did not alter PCNA levels in HEPA-C1C7, SKOV-3, B16, or 4T1-Luc2 cells. In contrast, the same treatment in the presence of serum reduced PCNA levels in all cell types except 4T1-Luc2 cells. These data were consistent with previous reports highlighting the ability of this ionophore to inhibit pro-proliferative pathways, including EGF-R, SUMO, and Wnt/β-catenin signaling [15,16,17,18,19]. It is, therefore, conceivable that monensin can produce a cytostatic effect in vitro only when cell proliferation is maintained by auxiliary exogenous pro-proliferative stimuli, such as those present in fetal bovine serum [27]. In the absence of these stimuli, monensin does not appear capable of influencing the intrinsic proliferative capacity of Hepa-C1C7, SKOV-3, or B16 cells.

Regarding TNBC 4T1-Luc2 cells, monensin did not affect PCNA expression even in the presence of serum. This observation appeared in contrast with a previous report of an antiproliferative effect of monensin in MCF-7 and MCF-10A breast cancer cells [25], where the cytostatic activity was evaluated as decreased cell colony formation. Such an effect, however, can be also visualized in our 4T1-Luc2 cell model (Figure 2d) and is likely to represent the reflection of a reduced cell density due to the killing activity of the ionophore.

Several factors can be instead involved in the different or delayed responsiveness of the other cell types to the effects of monensin on PCNA expression in the presence of FBS. Cancer cells in the absence of FBS undergo proliferation inhibition and apoptosis with differential timing depending on the cell-specific resistance to this condition [27]. Similarly, monensin might need dissimilar dosages to exert its cytotoxic action in different cancer cells (i.e., HCC cells [14] vs. TNBC cells). Thus, in the presence of FBS, each cell lineage might require differential concentration or time of treatment with monensin to manifest the effects on PCNA expression, and this might explain the observed difference (Figure 3).

The possible FBS components involved in this process are at the moment unclear. Dedicated methodological analysis would be necessary not only for their identification but also to establish alternative culture conditions able to ensure the long-term survival of cultured cells also in the absence of FBS. Our findings indicate, in fact, that the presence of FBS might represent a possible confounding factor when comparing in vitro and in vivo results related to proliferative processes.

Standard chemotherapy relays on a general cytostatic effect and chemotherapy treatments for TNBC patients inevitably interfere with physiological signaling pathways, often inhibiting necessary cell turnover in normal proliferating tissues and causing side effects that exacerbate the morbidities in TNBC patients [32]. These include a wide spectrum of adverse reactions during and after treatment, which, as reported by meta-analysis studies involving 4474 participants [33], results in serious symptoms, such as diarrhea, nausea, vomiting, hair loss, fatigue, weight loss, pain, and a decline in immune defenses [32,33].

The present study showed that monensin achieved its antitumor efficacy in mice TNBC through a sodium-dependent and cancer-specific cytotoxic mechanism, without altering the structural and functional integrity of healthy non-proliferating and proliferating tissues.

These observations point to the potential role of monensin as a therapeutic alternative to standard cytostatic treatments, without the detrimental side effects associated with their employment. Investigations with additional models and larger experimental studies will be, however, needed to further validate and more extensively analyze the anticancer effect of monensin against TNBC.

In this study we used immuno-competent mice and analyzed TNBC tumors derived from mice TNBC cells, to more closely represent physiological conditions. This model, however, could not differentiate the eventual and additional effects of monensin on the anti-cancer immune-inflammatory reactions. To overcome this limitation, it will be useful, in future research, to analyze the anticancer activity of monensin also in immuno-compromised mice models of TNBC. These models will also consent to evaluating in vivo the effects of monensin against TNBC developed from human TNBC cells such those used in vitro in the present study (Table 1) or in previous studies [19], thus increasing the clinical relevance of the findings.

The present study focused its investigation on a mice model of primary TNBC. Future research employing metastatic models of TNBC will be fundamental to extend the analysis of the anticancer activity of monensin to processes of main relevance for the treatment of the advanced stages of TNBC such as epithelial mesenchymal transition and migration TNBC cells and eventually metastasis formation.

Although mice studies have by now excluded adverse effects, three reported cases of severe human rhabdomyolysis after monensin ingestion [34,35], the variability of the toxic effects of monensin in different species [36], and its previously described cytostatic effects [15,16,17,18,19] might have, by now, prevented the direct employment of monensin in clinics.

Our findings on the specific anticancer activity of monensin unrelated to antiproliferative effects and the numerous previous reports on its potent anticancer activity [15,16,17,18,19] represent, however, a solid scientific base for further investigations aimed at overcoming all potential translation barriers. Such investigations, possibly including the development of novel and more active monensin analogs, will help to identify the lower dosages for a possible safe employment of sodium-ionophore compounds in the therapy of TNBC and of cancer in general.

## 5. Conclusions

TNBC is the most aggressive breast cancer subtype, with few therapeutic options, none of which are durable.

This study investigated the experimental hypothesis that TNBC cells owning an elevated intracellular Na^+^ level compared with normal cells could be selectively targeted and killed through a pharmaceutically induced Na^+^ influx. To this purpose we analyzed the anticancer activity of antibiotic cation ionophore monensin against TNBC using an immunocompetent wild-type mouse model of TNBC and murine TNBC cells. We showed that monensin effectively inhibited TNBC growth through a Na^+^-dependent and cancer-specific cytotoxic mechanism, not reliant on cytostatic activity.

While these findings were limited to cellular and in vivo mice models of TNBC, they underscore the potential of ionophoric compounds like monensin as innovative therapeutic agents for TNBC devoid of harmful effects on healthy proliferating tissues.

The results presented in this study additionally enlighten the relevance of investigating the high sodium content of cancer tissues as an innovative target in oncology. The main issue in cancer therapy is the extreme variability of each cancer subtype and the consequent lack of general and cancer-specific actionable markers. A new frontier in oncologic therapy is thus precision medicine [37], which, however, implies a complex and expensive development of personalized therapies.

The high sodium content in cancer is associable with two of the few features common to virtually all cancers: alkaline pH and aerobic glycolysis [9,10]. This indicates that the disruption of sodium homeostasis of transformed cells would likely affect not only TNBC in individual patients but all tumors in general. Future and deeper research, to be also extended to extra-breast tumors and aimed at solving all possible translational obstacles, is, however, needed for clinically applying this stimulating and, as of now, unique therapeutic possibility.

## Figures and Tables

**Table 1:** Na^+^-dependent ATP depletion and killing of murine and human TNBC cells treated with monensin. Murine 4T1-Luc2 (4T1) and human MCF7 TNBC cells were exposed up to 24 h with 1 µM of monensin. ATP and sodium content (evaluated as ING fluorescence) as estimated after 8 h treatment and cell viability measured at 24 h treatment with monensin are expressed as % controls (n = 3 independent experiments). * *p* < 0.005. Significance evaluated with O-way analysis of variance (ANOVA) Bonferroni multiple comparisons test.

	Viability (%)	ATP (%)	ING Fluorescence (%)
4T1 cells + M+ Na^+^	3 ± 2	51 ± 5	62 ± 4
4T1 cells + M- Na^+^	80 ±9 *	96 ± 7 *	18 ± 3 *
MCF7 cells + M+ Na^+^	5 ± 3	55 ± 4	57 ± 5
MCF7 cells + M- Na^+^	82 ±7 *	98 ± 6 *	17 ± 3 *

## Data Availability

The data presented in this study are available on request from the corresponding author.
